# The potential use of *Drosophila* as an in vivo model organism for COVID-19-related research: a review

**DOI:** 10.3906/biy-2104-26

**Published:** 2021-08-30

**Authors:** Eşref DEMİR

**Affiliations:** 1 Medical Laboratory Techniques Program, Department of Medical Services and Techniques, Vocational School of Health Services, Antalya Bilim University, Antalya Turkey

**Keywords:** *Drosophila melanogaster*, COVID-19 pandemic, SARS-CoV-2, human disease models, CRISPR-Cas systems

## Abstract

The world urgently needs effective antiviral approaches against emerging viruses, as shown by the coronavirus disease 2019 (COVID-19) pandemic, which has become an exponentially growing health crisis. Scientists from diverse backgrounds have directed their efforts towards identifying key features of SARS-CoV-2 and clinical manifestations of COVID-19 infection. Reports of more transmissible variants of SARS-CoV-2 also raise concerns over the possibility of an explosive trajectory of the pandemic, so scientific attention should focus on developing new weapons to help win the fight against coronaviruses that may undergo further mutations in the future. *Drosophila melanogaster* offers a powerful and potential in vivo model that can significantly increase the efficiency of drug screening for viral and bacterial infections. Thanks to its genes with functional human homologs, *Drosophila* could play a significant role in such gene-editing studies geared towards designing vaccines and antiviral drugs for COVID-19. It can also help rectify current drawbacks of CRISPR-based therapeutics like off-target effects and delivery issues, representing another momentous step forward in healthcare. Here I present an overview of recent literature and the current state of knowledge, explaining how it can open up new avenues for *Drosophila *in our battle against infectious diseases.

## 1. Coronavirus disease 2019 (COVID-19) pandemic: a landmark in the history of infectious diseases

Epidemics of infectious disease caused by viruses and bacteria have been haunting humanity ever since the very earliest settlements, claiming millions of lives and disrupting communities across the globe. Even today, communicable diseases like lower respiratory infections, malaria, HIV/AIDS, and diarrheal diseases still rank among leading causes of death in many parts of the world. And for over a year now, we have been confronted with yet another global pandemic, which is caused by a novel coronavirus commonly known as SARS-CoV-2. According to the most recent figures published by World Health Organization (WHO, 2021), there have been over 100 million confirmed cases of COVID-19, with a death toll reaching over 2 million since the beginning of the outbreak in December 2019–March 2021. It is predicted that many millions of people will become infected before an effective vaccine has been developed and a widespread immunization campaign can be implemented, which will continue to put healthcare centres and staff under heavy strain and result in further loss of life. 

In the face of an exponentially growing health crisis, scientists from diverse fields have been making every possible effort to identify the key genomic features of SARS-CoV-2 and clinical manifestations of COVID-19 infection, including acute pneumonia, respiratory distress, cytokine storms, and damage to multiple organs (Guan et al., 2020; Jiang et al., 2020). However, there are still several questions and challenges that needs to be addressed, which include factors influencing virus infectivity, mode of transmission, routes of infection, genes responsible for susceptibility and infection severity, protective role of immune system, and host-virus interactome (i.e. interactions between viral and host proteins). As new symptoms and complications related to COVID-19 infection continue to appear at different locations, we must constantly update our knowledge and provide guidance to reduce the uncertainty surrounding the infection and tackle the spread of current pandemic. 

The novel coronavirus SARS-CoV-2 responsible for COVID-19 pandemic actually belongs to *Coronaviridae* family consisting of positive-strand RNA viruses, which cause respiratory tract infections in mammals through direct cytotoxic effects and inflammatory reactions mediated by cytokines in the host immune system (Liu et al., 2020). So far seven coronavirus species have been identified to cause infection among humans, which include MERS-CoV and SARS-CoV. Since coronaviruses can evolve rapidly and be transmitted to humans from a wide range of animal hosts like rodents and bats known as high-risk viral reservoirs, scientific efforts should focus on developing new methods and weapons to facilitate the fight against coronaviruses that may undergo mutations in the future (Mohd et al., 2016). Although SARS-CoV-2 has been observed to mutate significantly more slowly as compared to HIV or influenza viruses (Callaway et al., 2020), a recent paper reported that more transmissible forms of SARS-CoV-2 with distinct genomic sequences are becoming the dominant forms across Europe (Korber et al., 2020). A new variant of concern, called lineage B.1.1.7, was reported in UK in December 2020, with enhanced transmissibility and potential to expand the predicted trajectory of the pandemic (Galloway et al., 2021). At present, the clinical outcomes of such variants appear to show no difference from that of the original strains, but we know that a higher rate of human-to-human transmission means more COVID-19 cases and greater number of people requiring clinical attention. This could further increase the burden on our already-strained health systems and ultimately lead to more deaths. Therefore, we need aggressive pan-coronavirus solutions to mitigate the spread of the virus, design therapeutic agents and effective vaccines, as well as diagnostic tools for widespread testing; otherwise global death toll could reach terrifying peaks in the coming months (Galloway, 2021).

## 2. In vivo model organisms in COVID-19 research

Although in vitro studies using human cell lines or organoids offer invaluable insight into the virus infection, viral life cycle, and replication, the intricate pathogenesis of COVID-19 disease may only be explained through recreating systemic virus-host interactions in animal models (Takayama, 2020). Ever since the outbreak of COVID-19 pandemic, several animal models have been employed in experiments to decipher the codes of the infection or to test potential antiviral drugs. A study, for example, used rhesus monkeys (*Macaca mulatta*) to understand whether primary infection could protect patients from the risk of reinfection, finding that the monkeys reexposed to the virus had no recurring COVID-19 infection (Bao et al., 2020a). Another study using nine adult rhesus macaques found that the infected animals developed a near-complete immunity against SARS-CoV-2 rechallenge (Chandrashekar et al., 2020). Even though higher mammals like monkeys and apes play a crucial role in enhancing our knowledge about human physiology and are still our key allies in solving puzzling problems related to infectious diseases like COVID-19, we should note that animal scarcity and ethical issues severely restrict the use of such models, with small samples often consisting of one or two monkeys. Naturally, this limits the generalizability of their results to wider populations or situations and complicates the interpretation of the data.

Mice are also among favourite animal models widely employed by virus researchers to identify pathological characteristics of viruses on account of their well-established immune system, breeding capabilities, and accessibility. However, a study from the early phase of the pandemic showed low angiotensin-converting enzyme 2 (ACE2)-binding affinity, one of the key determinants of infectivity, in mice and rats (Wan et al., 2020), thus limiting our ability to create an effective COVID-19 infection in such animals. On the other hand, some researchers used transgenic mice with expressed human ACE2 receptor and successfully infected these mice with SARS-CoV-2, observing virus replication in the lungs and reduced pulmonary function (Bao et al., 2020b; Winkler et al., 2020). While they suggested the use of transgenic mice as a suitable model organism for testing immunity and antiviral measures against COVID-19, the low supply of mice with humanized ACE2 receptors in laboratories due to the restrictions of the pandemic may limit its widespread use in experiments (Soldatov et al., 2020). In the search of alternative models, one study found that the golden hamster (*Mesocricetus auratus*) could be used in research on pathogenesis and transmission routes of SARS-CoV-2 (Chan et al., 2020). Ever since the discovery that ferrets can be infected by influenza virus by Smith et al. have been used as ideal animal models in influenza studies because they show symptoms similar to those of infected humans and they can transmit the virus to other ferrets (Smith et al., 1933). In this regard, Kim et al. studied SARS-CoV-2 transmission, replication, and viral shedding in a small group of ferrets (*Mustela putorius furo*) and reported certain limitations of this model, such as infected ferrets exhibiting only mild symptoms and lower viral loads in the lungs (Kim et al., 2020). Furthermore, recent studies have revealed that SARS-CoV-2 replicated poorly in domesticated animals including pigs, dogs, chickens, and ducks, while it could cause infection in cats through airborne transmission (Shi et al., 2020). However, the number of animals used in such experiments was rather low (as low as five or six animals) due to ethical concerns over euthanasia of healthy animals after the experiments. As for larger animals, Cynomolgus macaques (*Macaca fascicularis*), a species native to Southeast Asia, have been proposed as another suitable animal model in identifying critical pathways of coronavirus infections, since they were permissive to such infections and exhibited certain symptoms comparable to those associated with COVID-19 (Rockx et al., 2020). In an effort to develop protective or therapeutic measures against the explosive spread of the pandemic, scientists also carried out vaccine and drug trials in rhesus macaques to test whether such agents could treat or prevent SARS-CoV-2 pneumonia (Van Doremalen et al., 2020; Williamson et al., 2020; Yu et al., 2020). Even though such larger animal models appear to be the ideal choice in elucidating the key interactions between the virus and human hosts, their relatively slow reproductive capacity and growth rates tend to restrict their extensive use.

## 3. Potential application of *Drosophila* as a test model in viral infections

Due to growing ethical concerns and animal rights movements over the use of mammals like rodents or nonhuman primates in scientific experiments, researchers have been seeking out an ideal and simpler model organism that would allow them to circumvent the process of the ethics committee approval, which may be frustratingly difficult in some cases. In this regard, a species of fruit fly called *Drosophila melanogaster* has been gaining scholarly attention and acceptance as a potential *in vivo* model in recent biological, genetic, and medical research. In fact, this fruit fly might have become one of the best-known eukaryotic organisms in the world since it was the first complex organism whose whole genome was sequenced (Adams et al., 2000). A few years later, after the mapping of human genome was completed by the Human Genome Project in 2013, scientists began to identify homologies between the two genomes, which helped establish its role as a reliable model to study human disease processes. Today, discoveries related to human biology and genetics are described in fruit flies and then translated to mammalian organisms (Pandey and Nichols, 2011).

*Drosophila* offers a range of advantages over the use of mammals in experiments, including much lower production costs, a very rapid life cycle, high productive capacity, and far simpler genetics with only four pairs of chromosomes (Jennings, 2011). A female fly can produce up to 2000 genetically similar offspring during her lifetime (2 months), whereas most rodent models are able to generate only a handful of offspring every 12 to 16 weeks. In addition, about 75% of the genes playing a role in known human diseases have at least one functional homolog in the genetic code of *Drosophila*, so any new findings in the fruit fly often translate well into human health sciences (Lloyd and Taylor, 2010). The protein sequence similarity between *Drosophila* and mammals is normally at about 40%, though it could be as high as 90% in conserved functional domains (Pandey and Nichols, 2011). Above all, in spite of many obvious anatomic differences between humans and fruit flies, they share basic principles of cell biology, such as regulation of gene expression, formation of synapses between neurons, cell proliferation, differentiation into specialized cell types, cell signalling, apoptosis and autophagy. Furthermore, the response of these flies to various medications influencing their central nervous system is comparable to the effects observed in mammalian systems (Andretic et al., 2008). The immune signalling pathways that respond to cytokines are remarkably conserved from fly to man. Therefore, *D. melanogaster*, provides an excellent platform for studying the biology and function of cytokines. Categories of some human viruses studied using *Drosophila* as a model in vivo organism is shown in Table. Also, *Drosophila* as an in vivo model organism for SARS-CoV-2-related research potential is illustrated in Figure. A great deal of genes and pathways now subject to intense scientific investigation in humans and mammals were first identified in *D.* *melanogaster*. For example, the *Wnt *signalling pathway in mammalians was originally detected as *wingless* (*wg1*) recessive mutation in *Drosophila* leading to the development of wingless/halterless flies (Sharma and Chopra, 1976). At cellular level,*Wnt * signalling pathways are known to be involved in a wide range of human diseases and processes that regulate cell proliferation, morphology, and fate determination (Korkut et al., 2009).

**Table 1:** Categories of some human viruses studied using Drosophila as a model in vivo organism.

Name of virus/genome type	Diseases in human	Results	References
Human immune-deﬁciencyvirus (HIV)-1/single stranded RNA	Acquired immune deﬁciency syndrome (AIDS)	Inhibition of Toll pathway and induction of JNK pathway	Leulier et al., 2003
Severe acute respiratory syndrome coronavirus (SARS-CoV/single stranded RNA	A typical pneumonia	Possible interactions between the SARS-CoV3a with cytochrome c	Wong et al., 2005
West Nile virus (WNV)/single stranded RNA	West Nile fever (including meningitis and encephalitis)	Possible inhibition of RNAi in Drosophila by noncoding WNV RNA	Chotkowski et al., 2008
Inﬂuenza A virus (IAV)/single stranded RNA	Flu pandemics	Detected some host factors for inﬂuenza virus replication and host cell programming	Hao et al., 2008
Human cytomega-lovirus (HCMV)/double stranded RNA	Birth defects	Inhibition of embryogenesis via viral proteins	Steinberg et al., 2008
Epstein-Barr virus (EBV)/double stranded RNA	Infectious mononucleosis, several types of cancer, and multiple sclerosis	Determination of related with human suppressors which focused by the BRLF1 of EBV to induce tumorigenesis	Adamson and LaJeunesse, 2012

**Figure:**
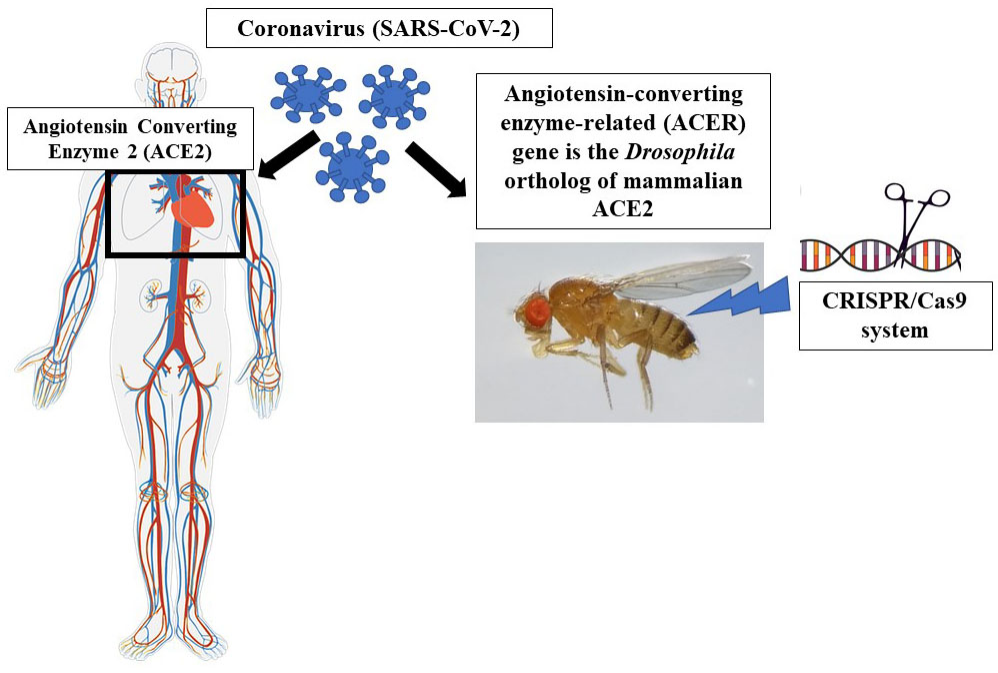
*Drosophila* as an in vivo model organism for SARS-CoV-2-related potential research.

In unraveling the codes of human viral infections, fruit flies have for some time been used as a model organism to study host interactions with known pathogens, which has taught us valuable lessons about the pathology of infection. *Drosophila* models offer rapid and simple genetics allowing a great number of studies to be conducted in a short time that is not possible in large vertebrates or mammals (Dionne and Schneider, 2008). With the help of such a suitable and established model organism already used in characterization of several viral restriction pathways (Wang et al., 2006; Zambon et al., 2006; Chotkowski et al., 2008), novel therapeutic agents designed to treat viral infections can be tested in vivo in a much easier, faster, and more economical manner. In fact, *Drosophila* has already been used to investigate the impact of various viral gene products, including HIV-Tat-related pathogenicity, effect of Vpu on fly immune defense, and chromosome number aberrations induced by HIV (Battaglia et al., 2001, 2005; Leulier et al., 2003). As known, Toll/nuclear factor-κB (NF-κB) signalling pathways are conserved between flies and mammals, Leulier et al. (2003) showed that a function for Vpu in the inhibition of host NF-κB-mediated innate immune defences and provide a powerful genetic approach for studying Vpu inhibition of NF-κB signalling in vivo. The HIV-1 Tat protein was first reported to bind to tubulin in fruit fly through the MAP-binding domain of tubulin (Battaglia et al., 2001) and this type of interaction where HIV-1 Tat protein binds tubulin and microtubules was later demonstrated in mammalian cells (Chen et al., 2002). In their review paper, Spresser and Carlson concluded that *D. melanogaster* could be used as a complementary model organism in research into genetics associated with HIV-1 infection (Spresser and Carlson, 2005).

Over the past decade,* Drosophila* has been employed by studies that investigate specific microbial virulence factors, molecular characteristics, and cellular functions of viruses relevant to human health (Hughes Amanda et al., 2012; Panayidou et al., 2014) and host antiviral immunity in different animal species (Xu and Cherry, 2014; Nainu et al., 2017). Given that all viruses depend on host cell proteins to complete their viral life cycle, accurate detection of host cellular factors associated with each step of SARS-CoV-2 replication can ultimately afford us a wealth of knowledge in designing therapies for COVID-19. However, this might take a long time with traditional genetic screening techniques and mammalian animal models. In this context, Hao et al. (2008) used a novel genome-wide RNAi screen in *Drosophila* to identify host genes playing a role in the replication of H5N1 and H1N1 influenza viruses. They modified influenza virus to create infection in fly cells and identified the gene expression of the virus, reporting that three *Drosophila* genes with human homologs had important functions in viral replication. Such an approach could accelerate the development of novel therapeutics and vaccines in the battle against future virus strains resistant to available remedies (Hao et al., 2008). Furthermore, previous work utilized GAL4/UAS (upstream activating sequence) system to characterize gene expression and function of SARS-coronavirus 3a protein in a transgenic *Drosophila* model (Wong et al., 2005). A similar study described the proapoptotic role of the SARS-coronavirus M protein in fruit flies (Chan et al., 2007). Adapted by Brand and Perrimon in 1993, the GAL4/UAS system has been a powerful tool used for targeted gene expression and function in *Drosophila*. The GAL4 system can thus be used to study regulatory interactions during embryonic development. In adults, targeted expression can be used to generate dominant phenotypes for use in genetic screens. In this landmark method, the gene that encodes the yeast transcription factor GAL4 is inserted into the fly genome to activate GAL4 expression, so researchers can directly observe the impact of gene expression on immune response pathways (Brand and Perrimon, 1993). The GAL4/UAS system contains two parts: GAL4 under a specific promoter in one transgenic fly line, and the target gene of interest under the control of the UAS in another fly line. When the two parts are in the same fly in the offspring of these flies, GAL4 binds to the UAS driving the expression of the target gene. Using this bipartite GAL4/UAS system, Yang et al. (2020) recently developed and characterized a transgenic *Drosophila* model to screen disease-causing SARS-CoV-2 proteins, which they reported could successfully single out potential treatments designed to target pathogenic viral proteins. They showed that expression of ORF3a, a protein unique to coronaviruses, in the central nervous system of *Drosophila* flies was capable of causing apoptosis and inflammation of the nervous tissue, suggesting ORF3a could be a key virulence factor and their ORF3a *Drosophila* model reflected the main features of SARS-CoV-2 infection reported in human patients (Yang et al., 2020).

## 4. Potential uses of *Drosophila* in COVID-19 research

While transgenic* Drosophila* flies can be infected with viral pathogens for experimental purposes in laboratory settings, the wild types are also constantly infected by bacteria or viruses in their natural environment (Ekström and Hultmark, 2016), therefore examining the immune responses and antiviral immunity in this model organism could help elucidate human inflammatory processes and enhance our understanding of antiviral defenses. Even though *Drosophila* fights invasive pathogens through a series of sophisticated defense reactions, it does not possess an adaptive immune system, which renders *Drosophila* an ideal candidate as a model to investigate further characteristics of innate immunity that could otherwise remain overshadowed by adaptive immune system. Its cell-mediated innate immune response against pathogens involves activation of relevant signal transduction pathways, generation of antimicrobial peptides and ROS, phagocytosis of microbial pathogens, and encapsulation of invading elements (Salminen and Vale, 2020). Many organisms including humans and other vertebrates are known to exhibit similar innate defense mechanism that constitutes the first line of defense in the fight against microbial pathogens like bacteria and viruses before the acquired immune system is triggered. Thanks to the remarkable homology between the *Drosophila* and human innate immune mechanisms, such as the Toll pathway (Zambon et al., 2006), it may well be used in the studies aimed at elucidating our immune response to SARS-CoV-2 infection. As revealed by early research, as soon as this virus travels through the lower respiratory tract, a strong innate immune response is triggered, and during this phase, patient’s immune system begins to produce proinflammatory cytokines that result in viral sepsis together with other severe complications (Costela-Ruiz et al., 2020).

Another area of COVID-19 research where*Drosophila* offers an immense potential in antiviral drug discovery might be identification of disease-modifying targets. To accomplish this, various novel genetic tools already exist in our toolbox, such as GAL4 drivers that can activate tissue-specific expression of transgenes, particularly those found in the trachea of the fly (Liu et al., 2003). Such strains can be utilized to drive interfering RNA units in the *Drosophila* trachea to specifically inhibit expression of genes with human homologs that have key significance for the physiology of respiratory airways (Roeder et al., 2009). A study using RNA interference, for example, investigated the impact of viral infection on gene expression in wild-type *Drosophila* in an attempt to determine candidate genes with possible antiviral properties (Cordes et al., 2013). Results of this study showed that differential regulation of genes associated with Toll and immune-deficient pathways, cytoskeletal development, Janus kinase-signal transducer and activator of transcription interactions, and a potential gut-specific innate immune response were found (Cordes et al., 2013). In addition, further studies that will establish the differential gene expression profile of SARS-CoV-2 in *Drosophila* might help us track down genes of interest and their functions in innate antiviral immune response (Chan et al., 2007; Yang et al., 2020). Such approaches offer prospects for determining and confirming essential components of respiratory function that may point to druggable proteins or nucleic acids, or drug targets, in the treatment of respiratory diseases like COVID-19 (Pandey and Nichols, 2011). There is, however, rather limited research on using *Drosophila* as a model organism to screen vaccine candidates or therapies for COVID-19, but we have all the necessary biological tools ready and available for such an endeavour, particularly including the revolutionary CRISPR gene-editing systems (Chan et al., 2007; Yang et al., 2020).

## 5. Potential use of CRISPR gene-editing systems against COVID-19

As soon as the causative viral agent of COVID-19 was discovered, many scientists engaged in gene-editing efforts shifted their focus on the global battle against the pandemic. The current challenge facing researchers from diverse fields of science and technology is how to optimize and streamline gene-editing systems in creating better solutions. A groundbreaking discovery in the field of genetics, CRISPR associated with Cas9 systems have recently gained huge recognition in the scientific community, as they can give rise to a variety of applications in gene targeting and genome editing (Jinek et al., 2012). The multifaceted CRISPR gene-editing system allows modifications in the genes of both microbial pathogens and hosts such as gene deletions or site-specific gene insertions, as well as investigation of mechanisms involved in the development of infection, hence a versatile weapon against viruses and bacteria that rapidly evolve resistance. In fact, CRISPR-based approaches have already shown huge promise in identifying new therapeutic targets for the *Plasmodium* parasites that cause malaria (Lee et al., 2019), characterizing key bacterial virulence genes causing tuberculosis (Singh et al., 2016; Choudhary et al., 2019), and programming nucleases to kill pathogenic bacteria like *E. coli* and *Salmonella *(Hamilton et al., 2019). More importantly, Bikard et al. (2014) showed that RNA-guided nuclease could be programmed to target antibiotic resistance genes in a major bacterial human pathogen (*Staphylococcus aureus*) that causes pneumonia, skin and bone infections, reporting that CRISPR-Cas9 antimicrobials also functioned in vivo to destroy the bacteria in mice. After their detailed review of latest research into applications of CRISPR-Cas9, Doerflinger et al. (2017) concluded that this gene-editing technology was a valuable tool to produce streamlined antimicrobials against drug‐resistant strains of bacteria and to kill such pathogens with their own weapons.

## 6. CRISPR-based diagnostic tools

Considering that the mechanism was initially developed in bacterial genomes as a technique to disable RNA viruses, it is only natural that it will be transformed into a valuable tool to create rapid diagnostic tools and antiviral therapeutics. PCR-based COVID-19 testing is currently the primary method of diagnostic procedures across the globe; however, it requires a relatively long wait for results, sophisticated equipment, and technical expertise, which calls for alternative testing options, as rapid and ultrasensitive population-wide screening is vital to controlling the spread of infection. Accordingly, a recent study provided a series of assay design options ideal for use with CRISPR systems in the testing of SARS-related coronaviruses, as well as detection of 67 virus species, including influenza and rhinoviruses (Metsky et al., 2020). Researchers combining CARMEN pathogen detection platform and CRISPR-Cas13 method (CARMEN-Cas13) managed to develop a mass testing technique that screens over 4500 crRNA-target pairs through a single array. Their novel method can identify newly discovered pathogens like SARS-CoV-2 and has the potential to reduce the cost of testing substantially (Ackerman et al., 2020). In this technology (CARMEN), nanolitre droplets containing CRISPR-based nucleic acid detection reagents self-organize in a microwell array to pair with droplets of amplified samples, testing each sample against each CRISPR RNA (crRNA) in replicate. The combination CARMEN-Cas13 enables robust testing of more than 4500 crRNA-target pairs on a single array (Ackerman et al., 2020). Such multifaceted and scalable CRISPR-based diagnostic approaches could transform selective COVID-19 surveillance efforts into mass testing programs and help alleviate the current strain on health systems, as lack of accurate and rapid population-wide testing for SARS-CoV-2 still remains a challenge. In this regard, a new study has proposed a rapid and sensitive assay that uses CRISPR-Cas12a activity to detect SARS-CoV-2 in patient saliva in 15 min (Ning et al., 2020). CRISPR diagnostics could also be used to augment PCR-based testing, as demonstrated by a recent CRISPR-Cas13a-based test developed to detect SARS-CoV-2 directly in nasal swab RNA that can be read through a mobile phone camera (Fozouni et al., 2021). These different advances using CRISPR-Cas systems underscore their great potential in the development of rapid and accurate tests for COVID-19 and other infectious diseases that may emerge in the future (Xiang et al., 2020).

## 7. CRISPR-based antiviral therapeutics

Efforts to design and develop CRISPR-based therapeutics for infectious disease by modifying susceptible host genes or virus genes regulating replication have recently gained a significant momentum. The potential of CRISPR-Cas9 gene-editing system as a promising tool in treating and preventing HIV infection was shown in early research (Ebina et al., 2013). Another study reported that CRISPR-Cas9 system could be adapted for an effective antiviral therapy in human cells by targeting the viral genes associated with life-long persistent infections caused by herpes simplex viruses (Wang and Quake, 2014). Certain CRISPR effectors could be reprogrammed to combat against viral infections, as previous research revealed that they could inhibit replication of viruses in infected mammalian cells (Yin et al., 2017; Ophinni et al., 2018; Wang et al., 2018). For example, Cas13a, guided by CRISPR RNAs, was programmed to target respiratory syncytial virus and influenza virus in human cells and shown to treat and prevent such infections, with possibility of mRNA-powered antiviral interventions (Bawage et al., 2018). In vivo applications utilizing CRISPR-Cas13 for targeting RNA viruses to mitigate their infectivity have been suggested to pave the way for novel antiviral treatments for a wide range of viral diseases, including influenza and COVID-19 (Freije et al., 2019). Meanwhile, a team of researchers has recently developed a prophylactic antiviral intervention, adopting a novel CRISPR-Cas13-based approach will attack all known coronavirus species, including SARS-CoV-2, to achieve an aggressive pan-coronavirus protection (Abbott et al., 2020). Initially performing experiments designed to target influenza A virus (IAV) in human lung epithelial cells, the team refocused their efforts on testing their approach, called PAC-MAN, in SARS-CoV-2, to join the global battle against COVID-19 pandemic. However, despite its worth as a molecular approach in vitro, the major challenge here is whether we can translate research into clinical practice for potential COVID-19 therapies or vaccines, for we urgently need an effective in vivo method to safely deliver it to target cells in the lungs (Abbott et al., 2020). To that end, suitable and versatile model organisms are required in preclinical trials to validate the immunogenicity induced by CRISPR-based therapies (Mehta and Merkel, 2020). Through experiments with a highly suitable in vivo model like *D. melanogaster*, for example, the efficacy of novel therapeutics developed by CRISPR genome editing can be swiftly investigated prior to testing in higher vertebrate systems.

## 8. Use of Drosophila as a model in CRISPR-based solutions

The rapid rise of gene-editing systems has stirred public and academic debate over the ethical implications of using such methods in mammalian models in terms of animal welfare, risks, and uncertainty (De Graeff et al., 2019), because some CRISPR-based applications may still have insufficient efficiency in gene targeting, which can lead to unintended genetic modifications or mosaic mutations (Mehravar et al., 2019). Although limited in amount, there has been some research into CRISPR-mediated in vivo genetic screens using different animal models, such as mice (Chen et al., 2015; Manguso et al., 2017) and zebrafish (Shah et al., 2015). However, these experiments often involve complex and lengthy procedures that may restrict the scope and efficiency of mutation analyses.

In this context, researchers can rely on a better candidate known for its suitability for genetic screens: *D. melanogaster*. It has been employed as a highly efficient in vivo model in endogenous CRISPR-based genome modifications, such as gene insertions, deletions, and precise sequence edits (Gratz et al., 2015). Previously, Bassett et al. (2013) reported that they developed an easy and efficient CRISPR-Cas9 method to induce and monitor the mutagenesis of target genes in *Drosophila* with an impressive 10-fold in efficiency. Several other studies confirmed the speed and efficiency of *Drosophila* model in CRISPR-based genome-editing systems (Kondo and Ueda, 2013; Ren et al., 2013; Gratz et al., 2014; Port et al., 2014; Gratz et al., 2015). Besides, more recent research has shown that tissue-specific genome editing with CRISPR system (tsCRISPR) can be successfully applied in *Drosophila* to keep mutations limited to intended tissue or group of cells (Meltzer et al., 2019). This type of high-efficiency targeted gene modification in *Drosophila* was achieved by utilizing the bipartite GAL4/UAS system in combination with CRISPR-Cas9 method (Gratz et al., 2014). The use of tissue-specific CRISPR in *Drosophila* offers great promise in facilitating high-throughput in vivo screening, along with a great potential to overcome the restrictions of currently available screening methods (Meltzer et al., 2019). Genome-editing efforts often cause off-target alterations that may change the expression of otherwise intact genes or disrupt vital coding regions, which might induce genotoxicity (Fu et al., 2013; Eid and Mahfouz, 2016). In addition, epigenetic CRISPR therapeutics has also been reported to induce serious off-target effects causing dysfunction in a series of biological pathways, including innate immune responses (Khan et al., 2016). Nevertheless, such unintended off-target activities should pose no great concern in *Drosophila*, for they can be easily monitored in vivo throughout several generations thanks to its short life cycle. 

One of the most critical challenges currently complicating CRISPR systems is the lack of efficient delivery methods that would transport CRISPR-Cas9 components into desired cells, and nanoparticles present a range of advantages over other delivery methods, which include high efficiency, fine-tuning of particle size, low immunogenicity and mutagenicity nanosized particles (Rahimi et al., 2020). In this regard, *Drosophila * once again stands out asa familiar and highly efficient model gaining popularity in recent research into nanoparticle toxicity and genotoxicity (Demir, 2020), and it has the potential to facilitate and accelerate the testing of nanobased CRISPR delivery methods. Besides, the long-term impacts of CRISPR-based therapeutics for viral infections like COVID-19 on several biochemical pathways conserved within humans can also be studied in *Drosophila* much faster than in other in vivo models. Huynh et al. (2020) showed that a CRISPR-based technology, Cas13 works efficiently in *Drosophil* a, both ex vivo and in vivo. Like Cas9, Cas13 uses a guide RNA (CRISPR-RNA, 57 aka crRNA) to identify its substrate, that is RNA rather than DNA. Cas13 may have far-reaching implications for simplifying diagnostics. To develop a fast test for COVID-19, the specific high-sensitivity enzymatic reporter unlocking (SHERLOCK) protocol, a recently developed Cas13-based diagnostic test for infectious diseases, can detect the virus in 50 min (Gootenberg et al., 2018; Kellner et al., 2020). In another study, CRISPR/Cas13 was also used to detect SARS-CoV-2 (Metsky et al., 2020). These studies promise that important potential of Cas13 as a diagnostic and therapeutic tool. Screening the efficacy of antiviral agents or vaccines in a whole living organism without ethical considerations allows quick selection of compounds with better safety profiles before conducting preclinical tests in relatively expensive mammalian models.

## 9. Future perspectives

As part of mass vaccination campaigns in an effort to tackle the escalating COVID-19 pandemic, multiple countries have been allowing antiviral vaccines under emergency use authorization, while multiple candidate vaccines are either being tested in humans or scheduled for preclinical evaluation. In the meantime, new variants of SARS-CoV-2 with unusually large number of mutations are constantly detected across the globe (Galloway, 2021), thus we urgently need flexible and easily adaptable technologies like CRISPR-based systems to develop effective pan-coronavirus solutions as a quick response to pandemics. Even if the intensive global effort currently put in COVID-19 research fails to produce a clinically viable therapeutic option for the current pandemic, CRISPR will emerge victorious in the battle against future viral and bacterial infections. Once the limitations like off-target effects and delivery issues have been resolved, CRISPR-based therapeutics may represent another momentous step forward in public healthcare. And with its versatile features suitable for multiple roles in rapid and accurate testing, *D. melanogaster* could become a strong ally in our fight against various infectious agents. Angiotensin-converting enzyme-related (ACER) gene is the *Drosophila *ortholog of mammalian ACE2 (Liao et al., 2014). As commonly known, mammalian ACE2 arranges cardiac contractility mainly, while *Drosophila* ACER arranges heart development during embryogenesis (Crackoweret et al., 2002). In addition, ACER also probably arranges the heart physiology in adult flies as it is expressed in the heart of *Drosophila *during development (Houardet et al., 1998). Strategies targeting the use of this ortholog gene can be developed using a variety of genome engineering techniques and thus valuable fly model can be developed and used for COVID-19-related research. Even though all scientific attention is currently directed towards creating quick solutions that will end the pandemic and bring life back to normal, we should note that the advances made in gene-editing technology as part of this process will be applicable to a multitude of infectious diseases and future pandemics.
